# Annotations

**Published:** 1897-10-02

**Authors:** 


### ANNOTATIONS.

### The Illness of Mr. Ernest Hart.

WE are extremely grieved to hear of the serious turn which has been taken by the illness from which Mr. Ernest Hart has been so patient a sufferer. It has for long been known to his friends that he was affected with glycosuria, and those who have watched him busying himself about the affairs of the British Medical Association while obviously suffering from serious illness have been as constantly drawn to express admiration for the nerve, the pluck, and the endurance of the man, as they have to sympathise with him in the unobtrusive patience with which his active mind has borne the growing bodily weakness which his malady has involved. For the last couple of months he has been suffering from an affection of the leg in addition to his other ailment. With characteristic energy, however, he has refused to give in. He has conducted business; he has, along with Mrs. Hart, entertained large parties of friends at his beautiful house at Totteridge, being wheeled into the garden, and making himself always the centre of conversation and animation; he has even driven into town on the Thursdays, and has been carried upstairs into his office on an invalid chair to see the Journal through on the days it went to press, yet all the time he has been suffering from so grave a condition that in the end he has had to submit to amputation of the leg. We are sure that all our readers will join with us in expressing the sincerest sympathy with Mr. Hart in his misfortune, and in the hope that he will soon be restored to health. It so happens that both the editors of THE HOSPITAL have been associated with Mr. Hart in journalistic work, and we have both had plenty of opportunity for knowing and reason for admiring his rare insight, and his wonderful power of getting to the centre of things and putting before his readers the very essence of what was going on. Mr. Ernest Hart has devoted himself to the success of the Journal, and we hope that when the members of the British Medical Association realise how, in addition to serving them well for so many years, their editor has stuck to his post even when overpowered by sickness, they will hasten to give to him some expression of sympathy and gratitude. On inquiring we are informed that, notwithstanding the serious nature of his illness, Mr. Hart continues to make satisfactory progress.

### Sanitation on the Riviera.

THE search for health which seems the work in life of so many people not infrequently leads them into curiously insanitary places, and especially is this the case with those who travel southwards in search of warmth and sunshine. Much has been done in the towns of the Riviera to give to the sanitary appliances in use such an appearance of conformity to sanitary principles as shall make them acceptable to English eyes, and in many of the larger hotels complete sanitary installations have been made, which, if the supply of water were as good as the display of pipes, would serve their purpose very well. But in the private houses which are let for the season, often at very extravagant rents, we fear the state of affairs is not only very bad, but in some cases even worse than in days before the travesty of "English sanitation," which is now palmed off upon the tenant, was introduced. A winter resident on the Riviera writes to the *Times* drawing attention to the easy gaiety with which rich people, who in England spend money freely to ensure good sanitary surroundings, accept houses on the Riviera all the arrangements of which are of the most shocking description. He describes how last year, on arriving there, he found a house of an expensive character in a very good situation. In the middle of it in the basement was a great pit. Inquiring into the object of this he was informed that it was the "fosse" or cesspool for the whole house; and, on further investigation, he found that the pipes that led into it from the closets were very large and of rough earthenware, with very imperfect joints, and all inside the house. Another house he found with the cesspool partly outside and partly under the house, and again the roughest possible pipes, coming down inside the house, and no syphon trap, an inch or so of water in the closet pan being all the protection from the admission of sewer air. The fact is, there is a stage in sanitary progress which is worse than no progress at all, and that stage comes when water-closets are introduced and yet the "fosse" or cesspool is retained. In the days of cesspools pure and simple, each house, however badly it might smell, had its own private and particular odours, and kept its drainage to itself. But when "English sanitation" was introduced, and gallons of water were poured down in place of the tiny dribbles of former days, the "fosses" were found to fill too often, their thrifty owners stood aghast at the expense of emptying them, and in too many cases connected them with the drains. Such houses are insanitary in the worst sense of the word, and of such, we fear, are too many of the houses offered to English and other seekers after health in the sunny south.

### Marriage and the Race.

THE frequent and extremely various suggestions which from time to time are poured into the ear of an attentive world with the object of demonstrating how the human race may be improved by regulating man's love affairs, and especially by choosing for him his lady love, are a perennial source of amusement, and all the more so from the gravity and seriousness with which they are propounded. The last of these that comes to hand—hailing as so many of them do from that land of freedom, the United States—is the proposal to divide the race by careful medical examination into three classes: (*a*) the perfect animal; (*b*) the perfect animal with some defect in family history; (*c*) all the rest. The author of this plan is evidently impressed with the extreme difficulty of enforcing celibacy on the unfit, recognising the unfortunate fact that the unfit are even more prone to matrimony and paternity than the rest of mankind. But he seems to think that if granted a wife of some sort man would be content; so, having divided his race into three classes, he would ordain that the members of each class should mate only among themselves! By this simple plan everyone would be made content, and while class (*a*) and class (*b*) would increase in strength, in stature, and in vitality, class (*c*) would gradually die out. Most ingenious! But suppose class (*c*) did not die out, but lived on as an inferior race; then, indeed, would be the fulfilment of the prophecy contained in that clever little book "The Time Machine," where we find the successors of man upon the earth divided into two distinct races, only dimly related to one another, one beautiful and basking in the sunshine, the other hideous and living underground. So do scientific fiction and fictitious science go hand in hand.

